# Changes in projectile design and size of prey reveal the central role of Fishtail points in megafauna hunting in South America

**DOI:** 10.1038/s41598-022-21287-0

**Published:** 2022-10-25

**Authors:** Luciano Prates, Diego Rivero, S. Ivan Perez

**Affiliations:** 1 Consejo Nacional de Investigaciones Científicas y Técnicas, Buenos Aires, Argentina; 2 División Arqueología, Facultad de Ciencias Naturales y Museo, Universidad Nacional de La Plata, La Plata, Argentina; 3 Instituto de Estudios Históricos/Centro de Estudios Históricos “Prof. S. A. Segreti”, Universidad Nacional de Córdoba, Córdoba, Argentina; 4 División Antropología, Facultad de Ciencias Naturales y Museo, Universidad Nacional de La Plata, La Plata, Argentina

**Keywords:** Anthropology, Archaeology

## Abstract

Fishtail projectile points are the earliest widespread projectile type in South America, and share chronology and techno-morphology with Clovis, the oldest North American projectile type. Both were temporally associated with late Pleistocene megafaunal extinctions. Although the elusive direct evidence of human exploitation of megafauna in South America had kept Fishtails out of the extinction debate, a recent paper showed a strong relationship between the temporal density and spatial distribution of megafauna and Fishtail projectile points, and proposed that this weapon was designed and used for megafauna hunting, contributing to their extinction. If so, this technology must be distinctly different from post-FPP technologies (i.e., early Holocene projectile points), used for hunting smaller prey, in terms of distribution and functional properties. In this paper, we explore the changes in projectile point technology, as well as the body mass of potential megafaunal prey, and show that Fishtails were strongly related to the largest extinct megafaunal species.

## Introduction

Fishtail projectile points (FPP) represent the earliest widespread lithic tool type in South America^1–5^. These points were initially defined as “Fell points” by Junius Bird who recovered a set of them in the late Pleistocene cultural layers of Fell’s Cave, in the southern tip of Patagonia^6^. This archaeological complex became widely known mainly because it is a few centuries later in age and shares some techno-morphological features with North American Clovis points^1,7^, the earliest widespread techno-complex of the whole Americas^8–10^. Fishtail has been traditionally defined as a barbless point with a broad triangular or lanceolate blade, convex edges, and rounded shoulders^6,7^. Although research in recent decades has shown significant morphological variation among FPP, most specimens share specific design features such as broad and thin blades, and usually fluted stems^7^. Both Fishtail and Clovis points are also often associated with megafaunal remains. The temporal and stylistic similarities of Clovis and Fishtail projectile points have led some researchers to reasonably propose that they represent the expression of the same cultural or technological phenomenon^1,7,11,12^, and also that both were linked with megafaunal hunting. Despite differing opinions, Clovis and, to a lesser extent, FPP have always been at the heart of the debate about the early peopling of the Americas.

In North America, the link between Clovis points and the hunting of megafauna is quite clear^13^ and has fueled a long and ongoing debate on the role of humans in the Pleistocene megafaunal extinctions^14–18^. Although this debate has not yet occurred in South America due to limited direct evidence of human exploitation of megafauna^19–21^, a recent paper gives FPP a firmer and more important role in the extinction debate^22^. Based on indirect evidence, and chronological and spatial correlations between FPP and megafauna, Prates and Perez^22^ proposed that these projectile points were the main weapons used for hunting megafaunal species. Particularly, they show that (1) FPP appear in southern South America at the same time, ca. 13 k years cal BP, and in similar terrains, open areas where megafauna’s density also reached the highest values, and (2) both FPP and megafauna had almost completely disappeared after 11 k years cal BP. These results suggest that the rapid and successful dispersal of FPP technology drove the high rate of human population growth during the late Pleistocene^23^ and, then, contributed—along with other environmental factors and indirect effects^24^—to the extinction of megafauna.

The use of FPP to hunt megafauna can be evaluated through hierarchical levels of evidence: (1) direct evidence (association at kill/butchery sites), (2) functional evidence (distinctive characteristics that make them specifically designed for hunting megafauna), and (3) correlational evidence (spatial and chronological correlation with megafauna). Assuming that finding direct evidence is not a necessary expectation of megafauna hunting^25,26^ and that correlational evidence already has been evaluated^22^, in this paper, we assess the functional characteristics of FPP for hunting megafauna. If humans were the main agent responsible for extinctions and they affected megafauna dramatically and suddenly after the emergence of FPP (ca. 13 k years BP)^22^, this technology must be seen as a true behavioral revolution^27^. Thus, we expect strong functional relationships between FPP and megafaunal hunting. If so, FPP must be distinctly different from other early projectile points not used for megafauna hunting, in terms of distribution and functional properties. In this paper, we address these issues to better understand the relationship between changes in projectile point technology and potential megafaunal prey (body mass over 44 kg)^14^ of human hunters. First, we estimate and compare temporal and spatial distributions of early projectile points reliably dated to the latest Pleistocene (ca. 13 k–11 k years cal BP) and early Holocene (11 k–8.5 k years cal BP) in the Southern Cone of South America (i.e., FPP, Tigre, Pay Paso, Tuina, Ayampitin and Patagonian triangular-shaped) where FPP are spatially concentrated. To do so, we use site distribution and radiocarbon dates together with Maximum Entropy Distribution Models (MaxEnt)^28,29^ and Summed Probability Distribution (SCPD) methods^30,31^. If FPP were designed specifically for hunting extinct megafauna^22^, from a chronological and geographic perspective we expect them to be replaced, after extinctions and all over their dispersion area, by other types of points less efficient for hunting large mammals (e.g., smaller and with less killing power). Then we explore the design characteristics of different types of early projectile points and their relationship with megafaunal hunting. For that purpose, we analyze morphometric and technical data of early projectile point types of the Southern Cone. Particularly, we assess the morpho-functional correspondence of the projectile points with the available potential megafaunal prey in different periods (latest Pleistocene and early Holocene) and regions (Pampas, Andes, and Patagonia). We measure design effectiveness (robustness and penetration), damage capability, lethality, and manufacturing cost of each point type^32–37^. If FPP was the key technological factor for humans to successfully hunt megafauna beginning at 13 k years cal BP, we expect, from a morpho-functional perspective, these points to show the highest capability for tissue damage. Our results reinforce the expectation that the changes in early projectile point technologies in southern South America were associated with the changes in the body mass of available prey. FPP were strongly linked to predation on extinct megafauna and, unlike early Holocene points, their designs were constrained by the requirements for preying on larger animals.

## Results

### Spatial distribution and changes in the temporal density of projectile point types

**Figure 1:**
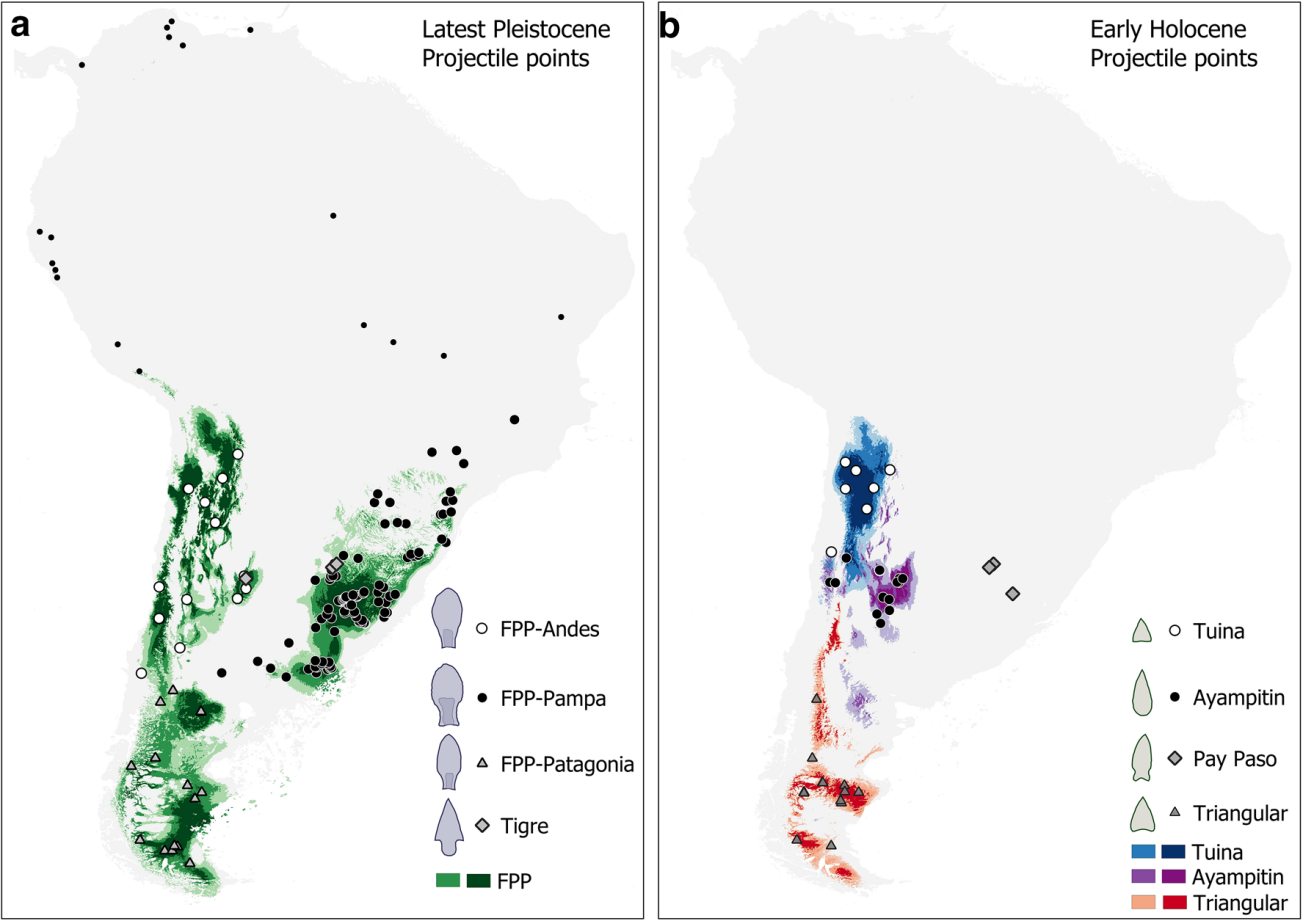
Spatial distribution of southern South American projectile points. Maps showing the potential distribution of latest Pleistocene (**a**) and early Holocene projectile points (**b**). Colors represent potential for distribution values above 0.6 (light colors) and 0.8 (strong colors). For Tigre and Pay Paso projectile points only the sites were graphed because of the small sample size. Northern South American sites with Fishtail projectile points also were graphed as small black points. Map generated with QGIS 3.16 'Hannover' (https://qgis.org/en/site/index.html).

The distributions of projectile points of the latest Pleistocene and early Holocene from southern South America were graphed using the spatial distribution modeling implemented in the MaxEnt method^28,29^. We considered projectile point records of ca. 13–11 k cal BP (latest Pleistocene) and 11–8.5 k cal BP (early Holocene), as well as the bioclimatic variables available in PaleoClim^38^ for each period. Figure 1 shows the spatial distribution of different types of projectile points for both periods. The predictive capability of the MaxEnt models was high for all types, with AUC values between 0.8 and 0.99 (FPP-Andes: 0.800; FPP-Pampas: 0.897; FPP-Patagonia: 0.973; Tuina: 0.978; Ayampitin: 0.797; Patagonian triangular-shaped: 0.989), suggesting that we obtain very good to excellent estimates of the distribution of each projectile point type. The distribution maps show that the latest Pleistocene FPP display high values of potential distribution and a large number of records in Pampas, with more dispersed occurrences in Patagonia; Tigre points are restricted to a few sites in Pampas. During the early Holocene, we observe that Patagonian triangular-shaped projectile point types present similar distribution to the FPP-Patagonia; Tuina and Ayampitin are superimposed on FPP-Andes, and Pay Paso occupy almost the same sites as Tigre points (Fig. 1).

**Figure 2:**
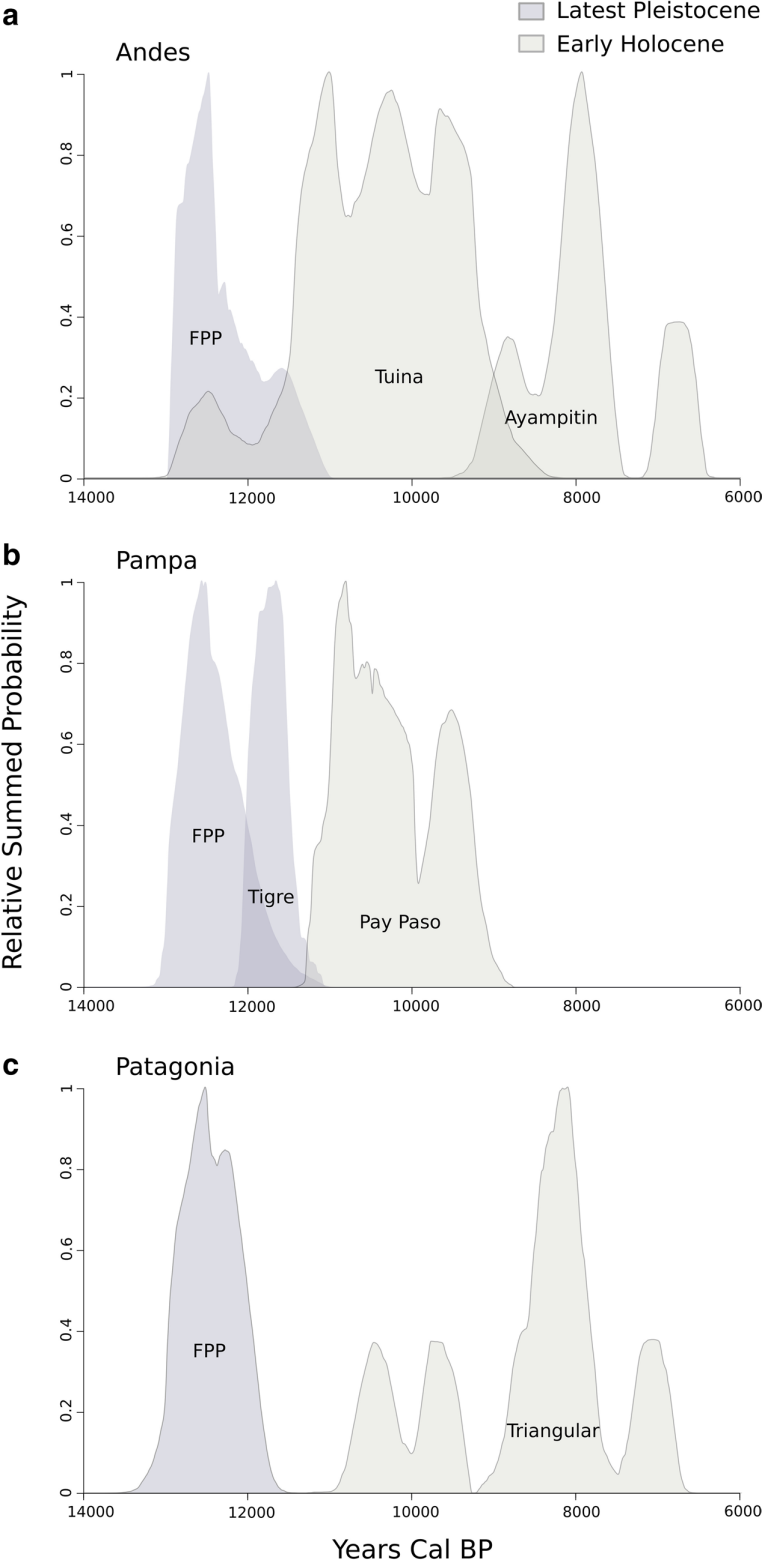
Summed probability distribution curves for three regions in southern South America. The temporal changes are displayed for Andes (**a**), Pampa (**b**), and Patagonia (**c**), using the estimated SCPD curve of each projectile point type or style. We only include projectile point types dated as older than 8.5 k years Cal BP.

The temporal changes in the density of the different types of projectile points were evaluated using radiocarbon dates and the Summed Probability Distribution method (SCPD method^30,31^). Figure 2 shows that changes in the density of dates of FPP are almost synchronous for the Andes, Pampas, and Patagonia, showing a clear increase after 13 k cal BP and a strong decline between 12.5 k and 11 k cal BP. Tigre points are contemporaneous with the last records of FPP in Pampas (Fig. 2). Early Holocene projectile types appeared after 11 k cal BP in the Pampas (Pay Paso) and Patagonia (Patagonian triangular-shaped), with later dates in the latter region. In the Andes, Tuina points partially overlap with FPP (Fig. 2). However, this superposition is due to a few old dates from a single site (Inca Cueva 4) that behave as outliers.

### Projectile point designs, capability for tissue damage and lethality

**Figure 3:**
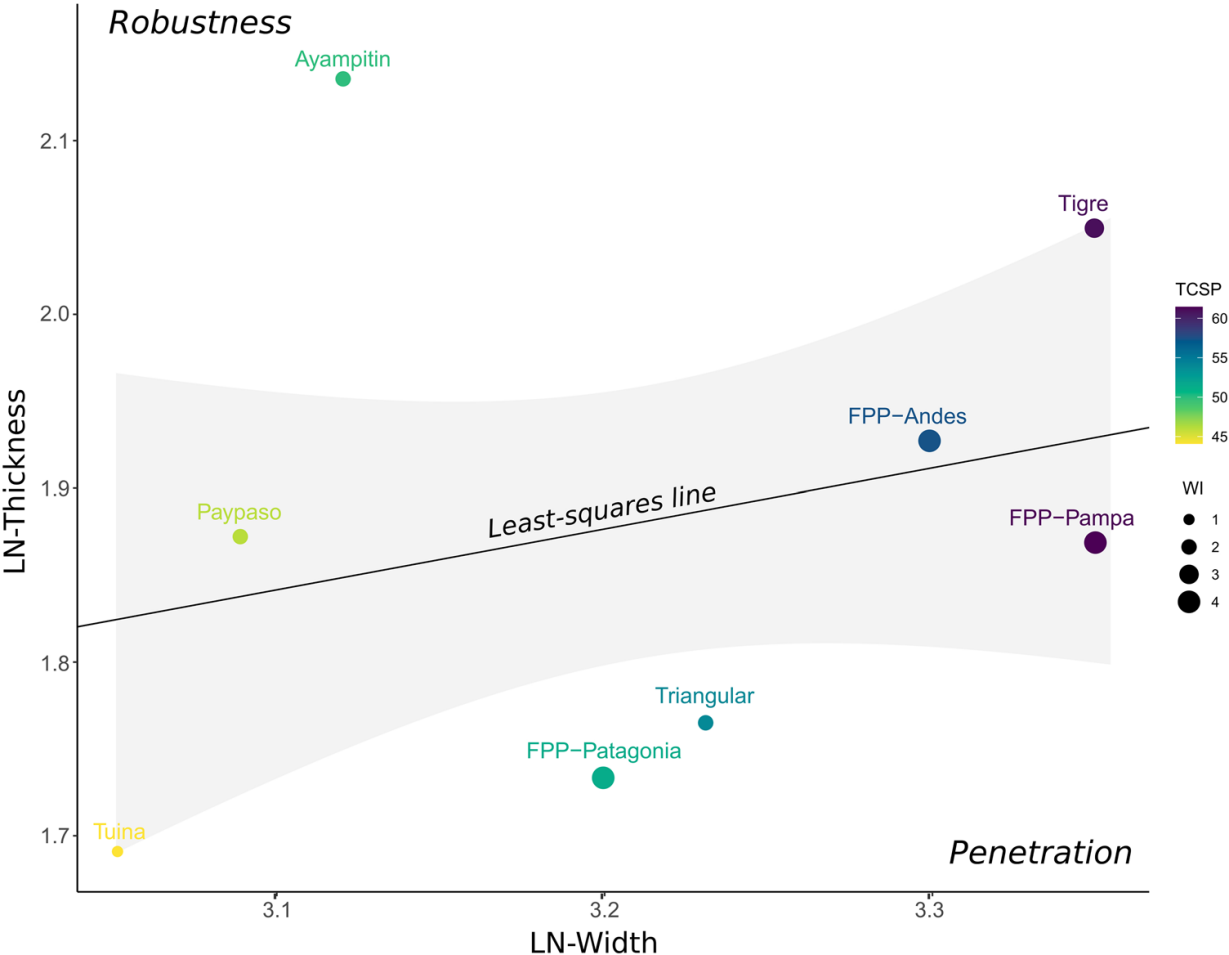
Scatter-plot of the relationship between thickness and width on a logarithmic scale of the six projectile point types separately. The solid line is the least-squares regression line of the data points representing the mean values for each projectile point type. The gray area represents the confidence interval of the least-squares line. Projectile points with means in the upper left above the regression line display designs that maximize robustness, and points in the lower right below the regression line display designs that maximize penetration. The tip cross-sectional perimeter (TCSP) and work investment (WI) are also displayed in color gradient (values expressed in millimeters) and ball size, respectively. See Buchanan and Hamilton^32^ for a similar approach to explore effectiveness and damage capability.

We explored the differences in projectile point design over time and geographical space using width and thickness^32^, the tip cross-sectional perimeter (TCSP, considered as a proxy for tissue damage)^33,34^, the wound surface area (WSA)^36^, and work investment in manufacturing (WI)^37^. Figure 3 displays the relationship between thickness and width on a logarithmic scale for mean values of the six studied projectile point types, considering separately FPP from Andes, Pampas, and Patagonia. The relationship between these variables was represented by the least-square regression line. By increasing the width of the projectile point, the potential injury caused to the prey increases; and by reducing the thickness, the penetration capability increases and also the risk of breaking due to loss of robustness. Therefore, it becomes necessary to compensate the width and thickness, so that the projectile point maintains the ability to penetrate, cause serious injuries and remain undamaged^32^. Because the least-square regression line was estimated in logarithmic scale, the relationship between both variables is exponential (Fig. 3).

Figure 3 also shows that the latest Pleistocene projectile points of the Andes and Pampas present the largest values of width, whereas the early Holocene points display the lowest values. The FPP for the Pampas and Andes are placed near the least-square line, suggesting that they were designed to maximize robustness and penetrating attributes^32^. FPP from the Pampas and Andes and Tigre projectile points show higher values of TCSP reflecting greater capability to generate tissue damage (Fig. 3). Early Holocene projectile points from Pampas and Andes present significantly lower values of TCSP than the latest Pleistocene ones (Table 1), suggesting a decreasing capability for generating tissue damage over time, even if this is balanced by increasing penetration of narrower points. This difference is not observed in Patagonia (Table 1), where Fishtail and early Holocene points (Patagonian triangular-shaped) show similar values of TCSP (Fig. 3). In the morphospace of Fig. 3, early Holocene point designs display a more random-like pattern—far from the least-square regression line—than the latest Pleistocene ones, suggesting that no one factor constrains the designs.

**Table 1:** Non-parametric mean differences tests among projectile point groups in TCSP index.

Region	Kruskal–Wallis test		Wilcoxon pairwise comparisons	
	Chi-squared	*P*		*p*
Andes	12.435	**0.002**	FPP—Tuina	**0.0195**
			FPP—Ayampitin	0.1217
			Tuina—Ayampitin	**0.0073**
Pampa	5.964	*0.051*	FPP—Tigre	0.7370
			FPP—Pay Paso	*0.0550*
			Tigre—Pay Paso	*0.0550*
Patagonia	0.009	0.923	FPP—Triangular	0.9400

The Kruskal–Wallis test evaluate the significance of TCSP index differences within geographical regions, whereas the Wilcoxon pairwise comparisons evaluate the significance of differences between all pair of projectile point types within each region.

Values in bold indicates significant differences and values in italics marginally significant differences.

The wound surface area (WSA), defined as the product of TCSP and the penetration depth^36^, measures the lethality of a shot into the thoracic cavity of an animal. This index was calculated for FPP, Tigre, Tuina, Ayampitin, and Pay Paso projectile points. Our results show that all of the late Pleistocene designs, except FPP from Patagonia, are potentially more lethal than those of the early Holocene (Table 2).

**Table 2:** Estimated mean and standard deviation (SD) TCSP, penetration depth and WSA of the FPP, Tigre, Tuina, Triangulars, Ayampitín and Pay Paso projectile points.

Projectile point TYPE	Mean TCSP (cm)	SD TCSP (cm)	Mean penetration depth (cm)	SD penetration depth (cm)	Mean WSA (cm^2^)	SD WSA (cm^2^)
Tuina	4.4	0.5	20.22	0.62	88.97	10
PayPaso	4.6	0.5	19.97	0.67	91.88	10.7
FPP patagonia	5.2	0.82	19.22	1	99.96	15.9
Triangulars patagonia	5.4	1.7	18.97	2	102.45	31.5
FPP pampa	6.1	2.1	18.09	2.6	110.40	38.6
FPP andes	5.6	1.3	18.72	1.7	104.85	25.2
Tigre	6.1	1.7	18.09	2.1	110.40	31.4
Ayampitin	4.9	1	19.59	1.3	96.03	20

The Penetration depth was calculated from the lineal formulae of TCSP versus penetration depth, based on the experimental results of Sitton et al.^39^. WSA was calculated as the product of TCSP by the estimated Penetration depth^36^.

Finally, to estimate the work investment (WI) (taken and modified from Aschero and Hocsman^37^) presented in Fig. 3, we assign a value ranging from 1 to 4 for different technical procedures (e.g. bifacial reduction or thinning), according to increasing work difficulty. FPP have the highest WI (score 4), followed by Tigre (score 3), Patagonian triangular-shaped, Ayampitin and Pay Paso (score 2) and, Tuina projectile points (score 1) (Fig. 3). Techno-morphological features of FPP (great width, minimal thickness, bifacial thinning, overshot flaking and fluting) must have demanded the greater investment of work during the shaping process. Lesser work is involved in the early Holocene designs, which required smaller blade widths and simpler bifacial reduction/thinning.

### Body mass of potential prey

**Figure 4:**
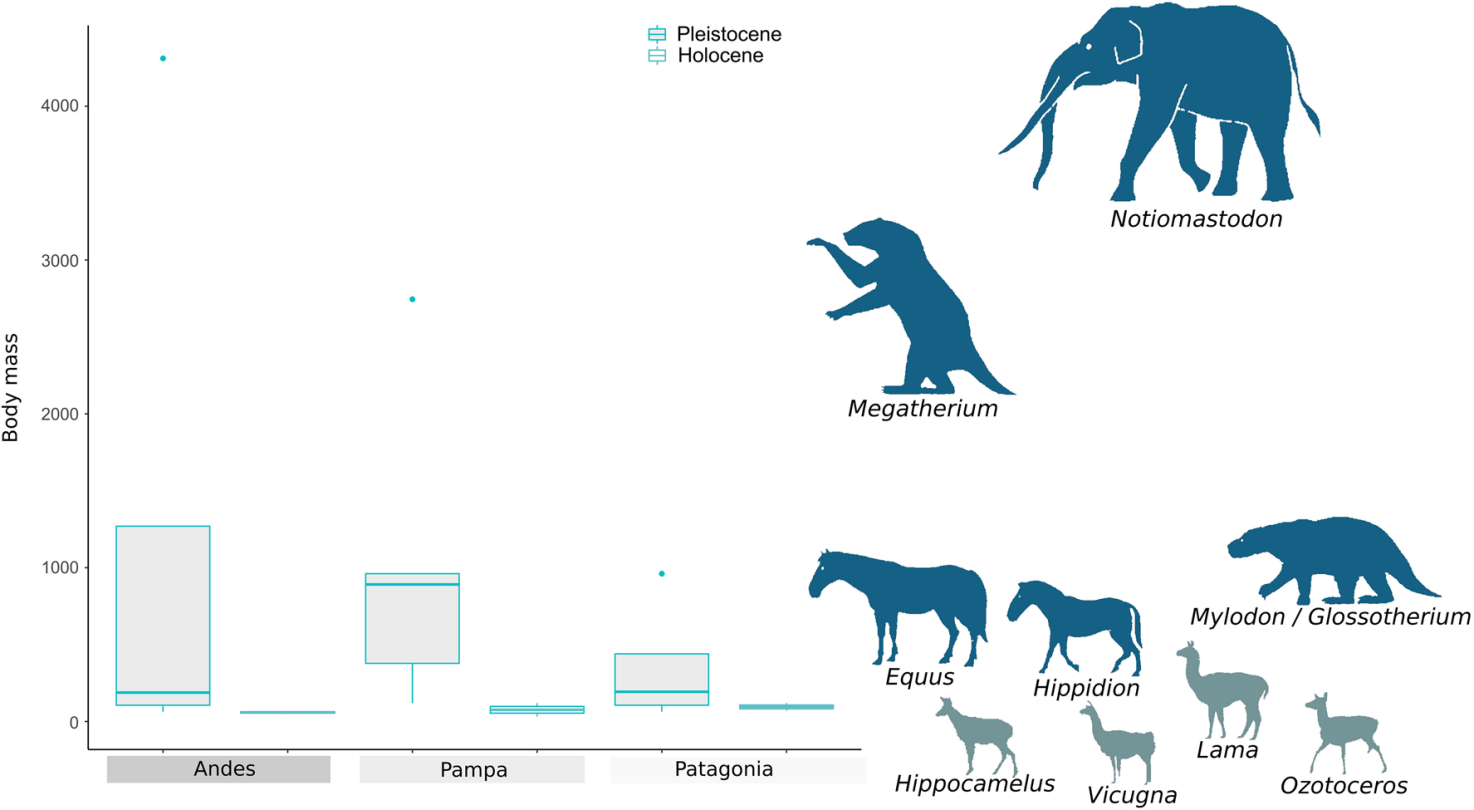
Body size variation in megafaunal species hunted by humans. Box-plots of body mass (in kg) of megafauna by region and period. The graph shows the minimum and maximum values (whiskers), the first and third quartiles (bounds of box), and the median (center line).

We described the body mass variation through time and space of megafaunal species found in archaeological contexts or strictly associated with humans using previous estimates based on regression models^40^. Figure 4 shows that body size values vary over regions. The mean of latest Pleistocene species in the Andes (*Notiomastodon platensis, Hippidion devillei, Lama guanicoe, Lama (vicugna) gracilis*) was about 1100 kg heavier than the early Holocene ones (*Lama guanicoe*, *Hippocamelus antisensis, Vicugna vicugna*). In the Pampas, latest Pleistocene species (*Megatherium (M.) americanum, Glossotherium robustum, Equus neogeus, Lama guanicoe, Mylodon darwini)* were about 950 kg heavier than the early Holocene ones (*Lama guanicoe* and *Ozotoceros bezoarticus*), and in Patagonia, the former (*Mylodon darwini*, *Lama guanicoe, Lama (vicugna) gracilis, Hippidion saldiasi*) were about 250 kg heavier than the latter ones (*Lama guanicoe* and *Hippocamelus bisulcus*). This pattern of body mass differences between latest Pleistocene and early Holocene fauna is similar to the above-described differences in projectile point design effectiveness (robustness and penetration), damage capability, and manufacturing cost, with large differences in Andes and Pampas, and small ones in Patagonia (Table 1 and Fig. 4).

## Discussion

### Projectile point diversity over time and space

Although non-preserved sharpened wooden spears or osseous projectile tips might have been used for hunting^41,42^, Fishtail stone points are the earliest widespread projectile point type in South America (13–11 k cal BP) and the only one with distribution in all latitudes and most environments, with the lowest density in the tropical lowlands and highest in the Southern Cone (Figs. 1 and 2). The higher densities are observed in those areas dominated by open environments of steppes or savannas at the end of the Pleistocene, especially Pampas and Patagonia^43^, where greater diversity and abundance of megafauna were also recorded^44^. If FPP were specifically designed for hunting extinct megafauna^22^, we expected that they would have been replaced after extinctions by other types of projectile points, smaller and less efficient for hunting large mammals, all over their dispersion area. As shown by Buchanan et al.^45^, larger North American Paleoindian points were used to hunt larger prey and smaller points were used to hunt smaller prey.

The expectation of FPP replacement after megafauna extinctions is fulfilled for the entire Southern Cone, in general, and for the three regions (southern Andes, Pampas, and Patagonia), in particular (Fig. 2). In all three regions, FPP appear around 13 k years cal BP, just before the growth in number and density of megafaunal species stopped abruptly and began a marked decline between 12.9 k and 12.8 k years cal BP^22^. From 12 k years cal BP onward, other types of projectile points appeared in the Andes (Tuina) and Pampas (Tigre and Pay Paso) which completely replace FPP around 11 k years cal BP, at the time megafauna almost disappeared. In Patagonia, although FPP disappear earlier than in the other regions (shortly before 11.5 k years cal BP), there is a period of around 500 years with no records of standardized projectile points. It is interesting that, in this region, after megafauna went extinct, the guanaco also retreated^46,47^ and the deceleration and decline in the growth of the human population seem to have been more drastic and longer than in other regions^23^. So, the prolonged invisibility of projectile points in the area also could be an effect of the low human population density and/or less availability of mammals after extinctions. Only around 11 k years cal BP middle-sized triangular-shaped projectile points appear in Patagonia and then persist in much of the region for several millennia (Fig. 2)^48^.

Although FPP are the earliest and most widespread projectile point in southern South America, they are not the only ones of latest Pleistocene age. There are several other types contemporary with megafauna, but with smaller spatial and temporal dispersion than that of FPP (Figs. 1 and 2). Firstly, Tigre points, with a restricted dispersion in the Pampas of Uruguay, show partial chronological overlap with the FPP and disappear together with them at ca. 11 k years cal BP. In a recent paper, it has been proposed that non-stemmed triangular points are probably contemporaneous with FPP in Pampas^49^, but we have not analyzed them here because they have not yet been securely dated. The only available dates (ca. 8 k years cal BP) come from Arroyo Seco 2 (Pampas) but these points were associated with human remains, mainly embedded in the skeletons^50^, and so, they were probably not used for hunting or at least they were not used exclusively for hunting. Conversely, the increase of the Tigre points temporally coincides with the decline of FPP (12 k−11.1 k years cal BP)^51^ (Fig. 2). So, it cannot be ruled out that they represent a local variant of points also designed for megafauna hunting, and that they fell into disuse and disappeared, as did FPP, after late Pleistocene extinctions.

A somewhat different case is that of the Tuina projectile points that spread over the southern Andes. Although Tuina points partially overlap in time with FPP (and with megafauna), the Pleistocene age of these points should not be considered reliable because the two oldest dates (coming from Inca Cueva 4) seem to be outliers. Tuina points, unlike Tigre, clearly increased in frequency during the early Holocene, after most megafaunal species had already gone extinct and after FPP disappeared (Figs. 1 and 2). This chronological trend fits well with the archaeological data which show that Tuina points are usually associated with extant species, especially *Lama vicugna*^52^. So, Tuina projectile points should not have been related to megafaunal hunting, but probably were used for hunting still extant camelids, both before (if the few outlier dates are valid) and after the Pleistocene extinctions.

Another latest Pleistocene projectile type, in addition to the Tigre and Tuina, but located mostly outside the study area, is the El Jobo lanceolate points, with a more northern and Andean distribution than that of FPP. Although ostensibly well-dated, El Jobo points are few and the oldest date from the Taima Taima site (ca. 16 k years cal BP^53^) is controversial^23^; they were probably penecontemporaneous with FPP. Beyond this chronological issue, the association between El Jobo points and megafauna [mastodon and megatherium]^54,55^ is strong and, hence, they could have been a local typological variant of spearheads used for killing this kind of prey. Nevertheless, since El Jobo points have only a slight geographical association with Pampean megafaunal species and overlap much more with northern species –especially proboscideans– than FPP, it is not clear whether El Jobo and FPP were synchronous geographic variants of projectile points with a similar function, or if the former were replaced by the latter. As experimentation by Frison^56,57^ showed, Clovis points (similar to FPP) effectively penetrated elephant hide, and Agate Basin points (more similar to El Jobo^58^) seemed better for punching through bison or cowhide. If so, effectiveness could explain the replacement of El Jobo by FPP.

### Morpho-functional properties of early projectile points

Our expectation was that, if FPP was the key technological factor for humans to successfully hunt megafauna beginning at 13 k years cal BP, these points should show the highest levels of functional performance. This is because larger animals are usually more difficult to hunt and kill, and require more efficient and lethal weapons than those used for smaller game^45,59^. The main function of the projectile point is to open a wound in the skin and tissue of the animal, to penetrate deep into the prey causing massive bleeding, and to reach some vital organ, thereby causing either rapid death or serious injuries that allow the hunters to pursue and finish off the wounded animal^32,45^.

**Figure 5:**
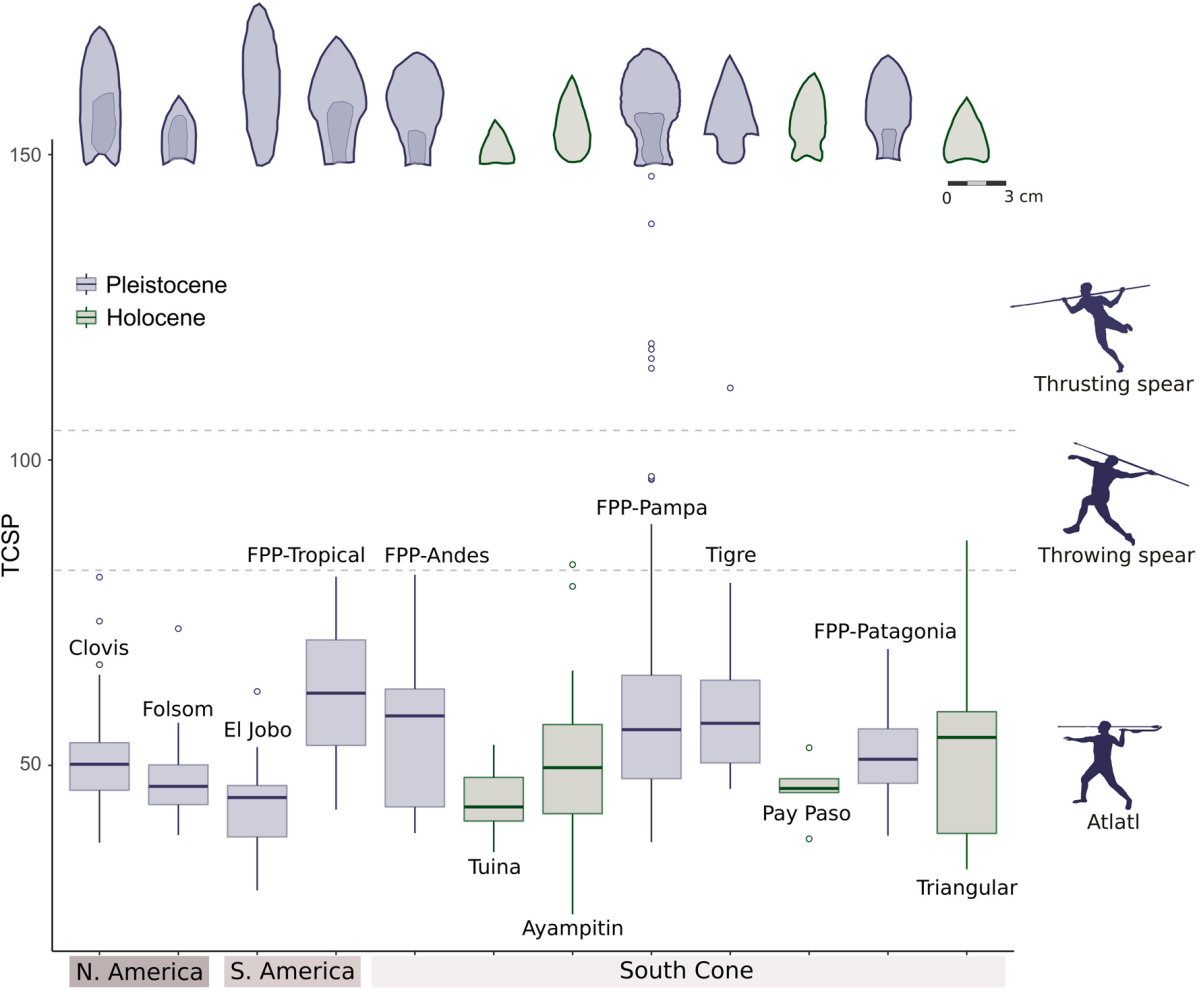
Box-plots of TCSP index by projectile point type (values expressed in millimeters), displaying the typical form of each type at the top of the x-axis. The graph shows the minimum and maximum values (whiskers), the first and third quartiles (bounds of box), and the median (center line). The functional assignment of projectile points is based on ethnographic and experimental data^60^.

Our results show that the oldest types of chipped stone projectile points of South America, in general, and FPP, in particular, are bigger, more harmful and lethal, and technologically more sophisticated than most of the early Holocene designs. As displayed in Fig. 5, the FPP from Pampas and Andes, and the Tigre points from Uruguay, all of them temporally and spatially associated with megafauna, are those with the highest values of TCSP and, hence, with the highest potential capability for tissue damage. The magnitude of the damage is mainly related to the maximum width of the projectile point, whose increase causes greater shock and faster bleeding of the prey^59^. Although the TCSP index is inversely correlated with the penetration capability, and reducing the size of the projectile point would increase penetration, this is limited by the need to cause injuries for rapid bleeding of the prey^60^. Therefore, it is essential to achieve a balance between achieving a TCSP small enough for adequate penetration, and large enough to produce a wound that bleeds easily^36,60^.

The Andes and Pampas FPP also offer a more efficient balance between width and thickness (represented by the least squares line in Fig. 3). As the point’s thickness decreases, robustness reduces and fragility increases, so that it becomes less resistant to the effects of mechanical forces acting at the moment of impact on the prey, such as buckling. Thus, as Buchanan and Hamilton^32^ have remarked for Paleoindian projectile points, it seems that FPP were designed to be long, wide, and thin, striking a balance between maximizing penetration power and minimizing the risk of the spearhead breaking^32^. Similar implications emerge from *wound surface area* analysis (Table 2) which clearly shows that the FPP technology is a highly effective design with a high damage capability. In general, points made before 12,000 years Cal BP would generate wounds 12% to 24% larger than Early Holocene ones.

By comparing the latest Pleistocene projectile technologies on a continental scale, which exceeds our study area, three additional trends emerge. Firstly, El Jobo points, the potentially oldest South American projectile technology, and one of the few types strongly associated with megafauna, have lesser damage capability than southern Tigre and FPP types (Fig. 5). If El Jobo was indeed the oldest South American projectile point type, they could have been used for hunting megafauna until being replaced by FPP, probably a new, more effective, and more reliable weapon for hunting large mammals. Secondly, the North American projectile points more clearly associated with megafauna (e.g., Clovis) would have had also larger potential for tissue damage compared to later North American projectile types (e.g., Folsom) (Fig. 5^59^). This pattern is similar to that observed in southern South America, but the values of damage capability of the North American points are less than those estimated for Tigre and FPP. It is also interesting to see how points seem to increasingly become more wide-bodied and narrow-stemmed southward (see Fig. 5) in contrast to the more straight-sided Clovis points.

Finally, variations in size and TCSP of projectile points could be related to their use in different weapon systems, as proposed by comparing projectile point TCSPs from ethnographic collections—whose weapon system was known—and experimental weaponry (Table IV in Hughes^61^). As shown by Fig. 5, most of the latest Pleistocene and early Holocene projectile types from both North and South America were probably used with atlatl darts^61^, as also proposed by experimental studies^62^. Only the largest types from the Pampas (Tigre but specially FPP) seem compatible with throwing or thrusting spears^63,64^. The latter weapon system is usually associated with hunting larger prey^65^ and would have been appropriate in regions where megafauna was more abundant and diverse. Although it has been proposed that Paleoindian projectile points could have been knives^12,66^, experimental studies of fracture show that Fishtail points were mainly used as projectiles^62^. Moreover, the fact that the primary function of a type of point is a projectile does not imply that it could not also have been used as a knife^67^.

In addition to the fact that latest Pleistocene projectile points from southern South America offered the highest levels of performance, their techno-functional properties also required a high degree of expertise and manufacturing cost (Fig. 3). FPP flintknappers produced points with great width and minimal thickness, using techniques including bifacial thinning, overshot flaking and usually fluting, which must have demanded a larger cost of work and more skill compared to that needed for producing any other projectile design^7^. Points with wide and thin blades would cause more tissue damage to prey, which could have compensated for the greater work and skill invested in their manufacture. It seems reasonable to us that such a specialized weapon would have replaced previous and less efficient designs (e.g., El Jobo), but would no longer have been produced once it became an unnecessarily expensive item after the prey for which it was designed (megafauna) disappeared. In fact, most of the early Holocene projectile point types, in addition to having less damage capability, demanded lower manufacturing costs and flintknapper skills than those necessary for producing FPP. Particularly, they have a lower damage capacity than Fishtail and Tigre points (Table 2 and Fig. 3) due to their smaller blade width, especially Tuina and Pay Paso types. Although most of the early Holocene types have robustness similar to that of the late Pleistocene ones, with a minimum thickness that favors penetration (Fig. 3), their damage capability is up to 20% lower (Table 2). As displayed in Fig. 3, the work investment in Ayampitín, Pay Paso, Patagonian triangular-shaped, and Tuina projectile points was limited to bifacial reduction/thinning, and smaller blade width production^68–71^.

Our expectation of better hunting performance of FPP compared to other designs is generally fulfilled, except Tigre points which present, together with the Pampa FPP, the highest damage capability values. This, along with the fact that Tigre points coexisted with megafauna and disappeared right after they went extinct, suggests these points could also have been used to hunt megafaunal species. Tigre points are thicker and have a lesser penetration capability than FPP (Fig. 3), as they present a less efficient relationship between width and thickness. They probably required less manufacturing costs and flintknapper’s skills than FPP due to the lesser work of thinning and the absence of fluting. Although the Tigre point might be only a local stylistic variant of latest Pleistocene points, its increase in frequency temporally coincides with the decline of FPP (12 k–11.1 k years cal BP)^70^ (Fig. 2) and with the decreased megafaunal abundance and diversity after 12 k years cal BP. So, an alternative explanation may be that the change in prey availability after 12 k cal BP made the high performance of FPP unnecessary and Tigre points were a more viable (and less expensive) technological alternative for hunting both megafauna and smaller prey.

**Figure 6:**
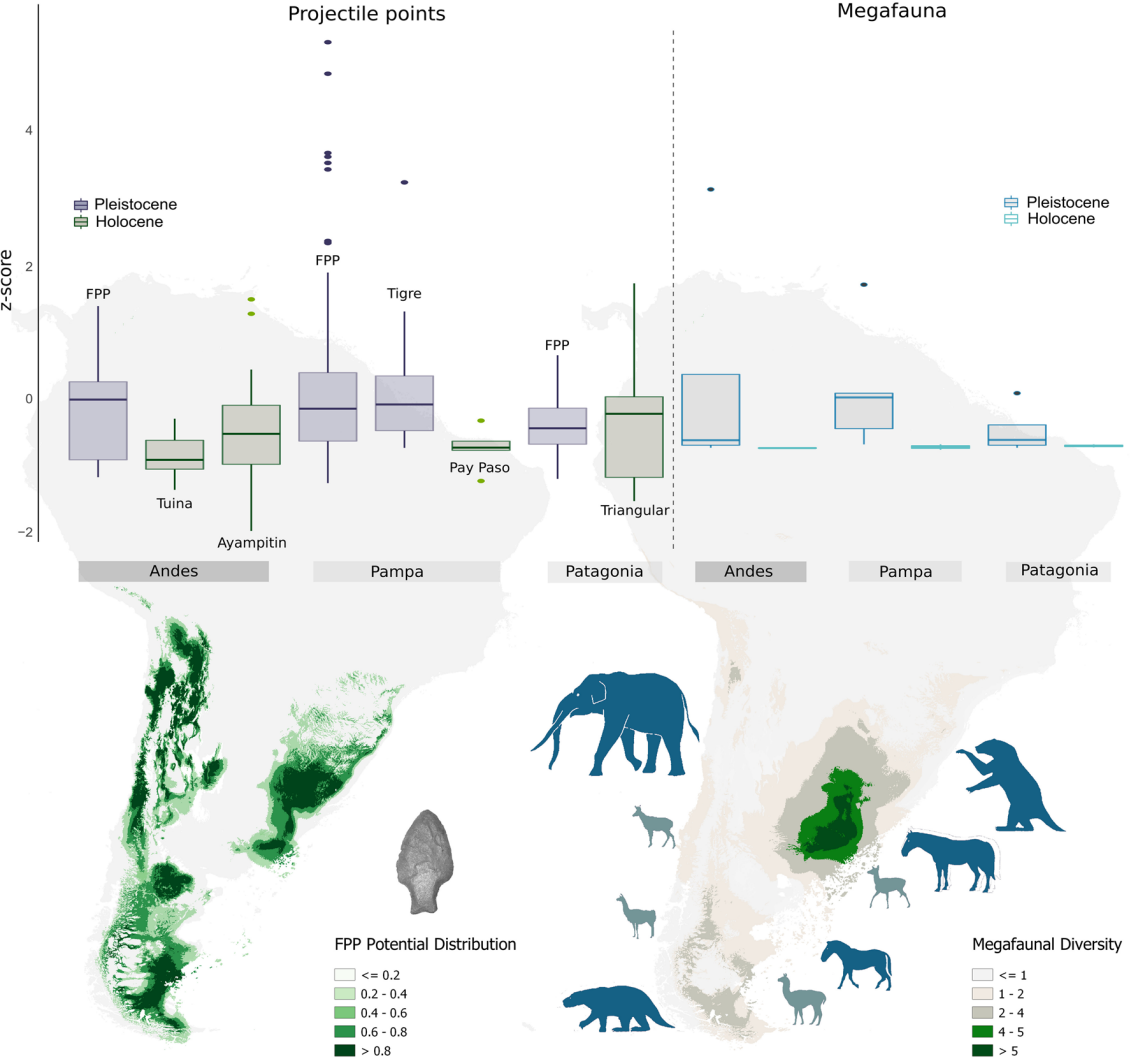
Box-plots of TCSP index by projectile point type and Body mass of megafauna by region and period, both data in z-score scale. The graph shows the minimum and maximum values (whiskers), the first and third quartiles (bounds of box), and the median (center line). Maps representing species diversity and projectile points distributions were generated with QGIS 3.16 'Hannover' (https://qgis.org/en/site/index.html).

The decreasing size of potential hunted prey in southern South America from latest Pleistocene to early Holocene is reflected in the decreased average body mass of mammals from archaeological sites (Fig. 4). If early projectile point types varied in concert with this change, not only is the capability for tissue damage of points expected to decrease after megafauna went extinct but also the magnitude of this decrease ought to be consistent with the magnitude of the body mass decrease within each region. As shown in Fig. 6 our results support this hypothesis since the reduction in the TCSP median values correlates with the decrease in the median body size of the regional fauna. The differences in TCSP values are significant or marginally significant for the Andes and Pampas, but not for Patagonia (Table 1), such as would be expected from the observed pattern in our results. Patagonian FPP show a relatively low damage capability, and also a low-efficiency adjustment between width and thickness, even when compared against the Patagonian early Holocene points (Patagonian triangular-shaped). Even so, this pattern is not necessarily contrary to expectations because the change in the body mass of prey after extinctions in Patagonia seems to have been not so marked, entailing only small differences in the mean size of prey between periods. This is probably because latest Pleistocene Patagonian megafauna was relatively smaller compared with Pampean and Andean, and also because early Holocene potential prey (*Lama guanicoe* and *Hippocamelus bisulcus*) were relatively bigger than those from the Andes or Pampas.

*In conclusion*, since the mid-twentieth century, Fishtail projectile points have been inextricably linked with the debate over the earlier peopling of South America; mainly because of their early chronology, their morphological affinity with Clovis points, and their temporal overlap with megafauna. Unlike in North America, the scant evidence for human exploitation of megafauna in South America has virtually nullified the debate on the relationship between humans and latest Pleistocene extinctions. Although a recent study showed a strong correlation between extinctions and the rise of Fishtail points and inferred human population^22^, the functional correlation between Fishtail points and megafauna hunting had not been evaluated so far. Here we have shown, first, that changes in morpho-functional properties of early South American projectile points varied in space and time in relation to the body size of the hunted prey. Second, that Fishtail points were not only temporally and spatially associated with extinct megafauna, but also that they were the most efficient technology to prey on them due to their greater capacity for tissue damage, damage/penetration ratio, and lethality. Third, that the greater efficiency of Fishtail points also made them technologically more expensive (due to bifacial thinning, overshot flaking, and usually fluting). Fourth, that Fishtail represented a new and revolutionary technology, probably introduced from the north^7^ that was better and specially designed for hunting megafauna. Fifth, that after the extinction of megafauna, Fishtail points were replaced in the different regions of southern South America by other types of points that had less damage capability, damage/penetration ratio, and lethality but were technologically less expensive, even though efficient enough for killing the smaller available prey. Finally, although direct archaeological evidence of human exploitation of megafauna remains elusive in South America^19^, our results reveal the central role of FPP in megafauna hunting and reinforce the hypothesis that humans had a direct and significant effect on its extinction. Future work is needed to clarify how resharpening affected the functional properties of FPP, where FPP technology came from, and to evaluate the potential association between hunt strategies and weapon systems and the hunting of different kinds of prey (in size and ethology).

## Materials and methods

*The spatial distribution of different types of projectile point types* of the latest Pleistocene and early Holocene from southern South America was estimated using the maximum entropy modeling approach implemented in the MaxEnt method^28,29^. This approach is widely used in paleoecology and archaeology because it is robust and based on presence-only data^28,29,72,73^. The dataset of projectile points used in the analyses consists of records with absolute dates—or highly reliable relative dates—and precise geographic locations. These data were obtained from original publications and some recent compilations (Data S1) and, although it may reflect biased research effort and/or systemic factors affecting the visibility of the archaeological record, the representativeness of the sample is not compromised. For the MaxEnt analyses, we considered separately records for the different types of projectile points assigned to the latest Pleistocene (ca. 13 k and 11 k cal BP) and early Holocene (ca. 11 k and 8.5 k cal BP), as well as the geographical variants of FPP, and used as predictors the bioclimatic variables available in PaleoClim for each period (http://www.paleoclim.org/)^38^. The performance of the models estimated for each projectile point type was assessed using the AUC statistic (i.e., area under curve)^28,29^, which measures the fit of the model varying between 0 and 1 (the better predictive value), with values below 0.50 indicating that the model is no better than the ones obtained by chance and values above 0.80 showing very good or excellent fit. All maps of potential distribution were graphed in QGIS 3.16.4 'Hannover'^74^.

*The temporal changes in the density* of the different types of projectile points were evaluated using radiocarbon dates and the Summed Calibrated Probability Density method (SCPD method)^30,31^. The radiocarbon dates for the FPP used were compiled by Prates and Perez^22^ and the dataset was complemented with dates for the other projectile point types obtained from archaeological publications (Data S2). This radiocarbon dataset was generated using the standard validation criteria proposed for archaeology, as summarized by Prates et al.^23^. The radiocarbon dates were calibrated using the Southern Hemisphere SHCal 20 curve. The SCPD curve was estimated for each projectile point type using calibrated dates binned by site in intervals of 200 years, and then smoothed using a moving average with window size of 500 years. The radiocarbon dates were calibrated and the SCPD curve was estimated with the package *rcarbon* for the R software 4.0^75^. Because we were interested in projectile technology occurring in times around megafaunal extinctions, we restricted the analyses to projectile point types whose earliest record was dated as older than 8.5 k years Cal BP.

The projectile point designs and capability for tissue damage were explored using morphometric and technical data (Data S3). We obtained metric data (width and thickness) for 127 FPP from southern South America and another 303 early points. Projectile points included in the analysis were complete or exhibited fractures that did not prevent the measurement of their width and thickness. Those with blades significantly affected by reactivation were excluded from the database (Data S3). This was because, after the reduction of the width of these points, their main function of causing massive bleeding could no longer have been properly fulfilled, and so they were probably discarded or repurposed as knives, as has been suggested for FPP with asymmetric blades^63^. We describe the differences among projectile point types and geographical variants of FPP using width and thickness^32^, the tip’s cross-sectional perimeter (TCSP^27,33,61^), and work investment in manufacturing (WI)^37^. We first explored the relationship between thickness and width of the six projectile point types—considering separately Andean, Pampean, and Patagonian Fishtail projectile points—on a logarithmic scale, using the least-square regression method. This analysis was performed following Buchanan and Hamilton^32^, who use the classical allometric approach to explore the differences in robustness and penetration of projectile points. Secondly, we explore the differential capability for tissue damage using as a proxy the TCSP^27,61^*.* The TCSP comes from ballistics and, calculated from the maximum width and thickness of the projectile point blade (TCSP = 4 × √[width/2]^2^ + [thickness/2]^2^), can be considered a proxy, directly proportional to its damage capability^27,33,61^. We also calculated the WSA index (wound surface area = TCSP × penetration depth) with the aim of estimating the lethality of the points^36^. The penetration depth capability for each type of projectile point was calculated according to the experimental data for the relationship between TCSP and penetration depth, obtained by Sitton et al. (Fig. 4)^39^.

The variation in TCSP and WSA for each projectile point type was compared with the differences in body mass estimation for megafaunal species^40,76^ associated with humans in the archaeological record from different regions (Andes, Pampa and Patagonia) and periods (latest Pleistocene and early Holocene). Finally, the estimation of the work investment (WI) for each projectile point type was based on Aschero and Hocsman^37^. We assign a value ranging from 1 to 4 for different technical procedures (e.g. bifacial reduction or thinning), according to the work difficulty, in increasing order. The work investment was ordered as follows: if making the projectile point included bifacial reduction (score 1), bifacial thinning (score 2), overshot flaking (score 3), and fluting (score 4).

## Supplementary Information

Supplementary Information 1.

Supplementary Information 2.

Supplementary Information 3.

## Data Availability

All relevant data are within the paper and its Supplementary Data files.
